# Alveolar ridge preservation with autologous particulated dentin—a case series

**DOI:** 10.1186/s40729-017-0071-9

**Published:** 2017-03-30

**Authors:** Silvio Valdec, Pavla Pasic, Alex Soltermann, Daniel Thoma, Bernd Stadlinger, Martin Rücker

**Affiliations:** 1 Clinic of Cranio-Maxillofacial and Oral Surgery, Center of Dental Medicine, University of Zurich, University Hospital Zurich, Plattenstrasse 11, 8032 Zürich, Switzerland; 2 Institute of Surgical Pathology, University Hospital Zurich, Zurich, Switzerland; 3 Clinic of Fixed and Removable Prosthodontics and Dental Material Science, Center of Dental Medicine, University of Zurich, Zurich, Switzerland

**Keywords:** Alveolar ridge preservation, Particulated dentin, Autologous augmentation, Bone augmentation, Bone substitute

## Abstract

**Introduction:**

Ridge preservation can be performed with autologous bone, alloplastic bone substitute material or a combination of both. Dentin is similar to bone in its chemical composition. In its use as bone substitute material, it undergoes a remodelling process and transforms to bone. The presented case report introduces a technique in which the extraction socket is augmented with autologous, particulated dentin.

**Material and methods:**

The fractured, non-savable mesial incisor of the upper jaw was carefully extracted in axial direction. After the extraction, the tooth was cleared from remaining periodontal tissue. The vital pulp tissue or a root canal filling, enamel and cementum were also removed. Following the particulation of the remaining dentin in a bone mill, the dentin particles were immediately filled orthotope into the alveolar socket. The soft tissue closure was performed with a free gingival graft of the palate.

**Results:**

After an observation period of 4 months, an implant was placed in the augmented area, which osseointegrated successfully and could be restored prosthodontically in the following. The results of this method showed a functional and aesthetic success.

**Conclusion:**

The pre-implantological, autologous ridge preservation with dentin could be performed successfully. For the establishment of dentin as augmentation material for jaw augmentation procedures, a prospective, clinical trial is now necessary.

## Background

Subsequent to tooth extraction, a resorption of the host bone as defined by atrophy of the alveolar ridge can be observed. Sutton et al. classified the different degrees of alveolar ridge atrophy [32]. Bone resorption especially occurs in the frontal and premolar area of the jaw in the region of the thin buccal lamella. This may lead to a change in contour [11, 28]. Physiological reason for this atrophy is the periodontal ligament blending into the bone. Overall, a total clinically relevant loss of bone height of approximately 2–5 mm in the first 6 months can be observed in the vertical dimension [10, 20]. After 12 months, the alveolar ridge may lose up to 50% of its width. With regard to dental implants, this implicates that an implant insertion in a sufficient bone bed will often not be possible. In order to prevent this bone atrophy, different methods of alveolar ridge preservation have been described. The augmentation of extraction sockets with deproteinized bovine bone is clinically well established and has analysed in various studies [17, 18, 31]. Systematic reviews showed a preservation of the bone contour for this method [6, 15].

Today, clinical techniques like the socket-shield technique are performed [9]. Applying this technique, a vestibular slice of the tooth root is left in the alveolar socket during tooth extraction. The reason is to prevent the resorption of the vestibular bony lamella. Studies show the osseointegration of implants having been inserted in such areas, thus indicating the biocompatibility of autologous tooth material [8, 13, 16]. The application of autologous dentin as a bone substitute for alveolar augmentation may serve as an alternative to the usage of xenogeny biomaterials. The chemical properties of dentin show a close relationship to bone and demonstrated a good osseous regeneration in an animal model [9].

Aim of this case series is to demonstrate the augmentation with autologous dentin as an interesting alternative to the application of xenogeny grafts.

## Material and methods

### Clinical technique

Four patients between 36 and 65 years of age are presented in this case series. There was no financial compensation. All four patients suffered from a trauma, causing damage to one or two teeth of the anterior maxilla. The frontal tooth/teeth has/had to be extracted. The pulp of the extracted teeth of three patients and the root canal filling of one patient had to be removed. All patients were informed on the operative procedure and possible risks and signed an informed consent. Treatment options were discussed.

**Fig. 1:**
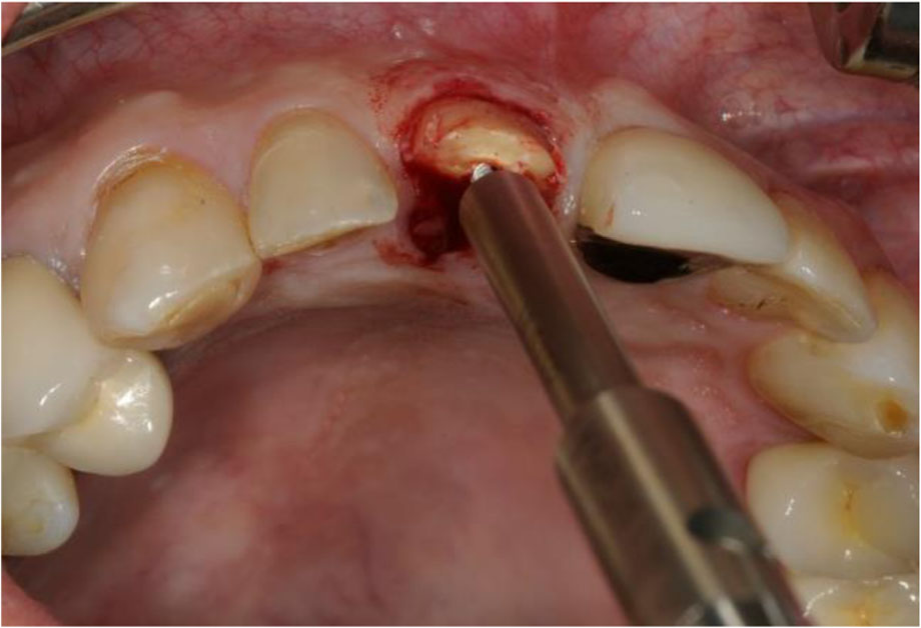
Extraction with the benex system

**Fig. 2:**
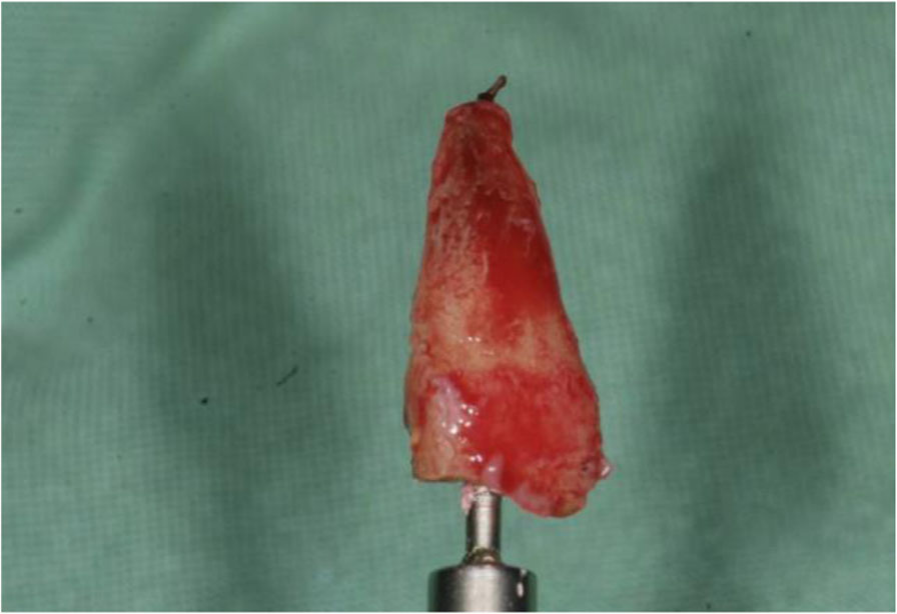
The remaining root of tooth 11

After mouth rinsing with a chlorhexidine solution (Chlorhexamed® FORTE 0.2%, GlaxoSmithKline Consumer Healthcare GmbH & Co.KG, Bühl, Germany), local anaesthesia (4% Ubistesin® with 1: 200,000 adrenaline, 3M Espe AG, Seefeld, Germany) was applied. The tooth extraction was performed carefully using a special extraction-system (Benex II extraction-system, Helmut Zepf medical technology GmbH, Seitigen-Oberflacht, Germany) in order to preserve bone and soft tissue (Figs. 1 and 2).

The clinical and radiographic examination showed healthy periodontal structures; the buccal wall was intact without fenestration with a minimal thickness of 1 mm; the discrepancy between the buccal height of the socket and the palatal height was not more than 3 mm; and the socket was within the bony envelope in all four cases.

**Fig. 3:**
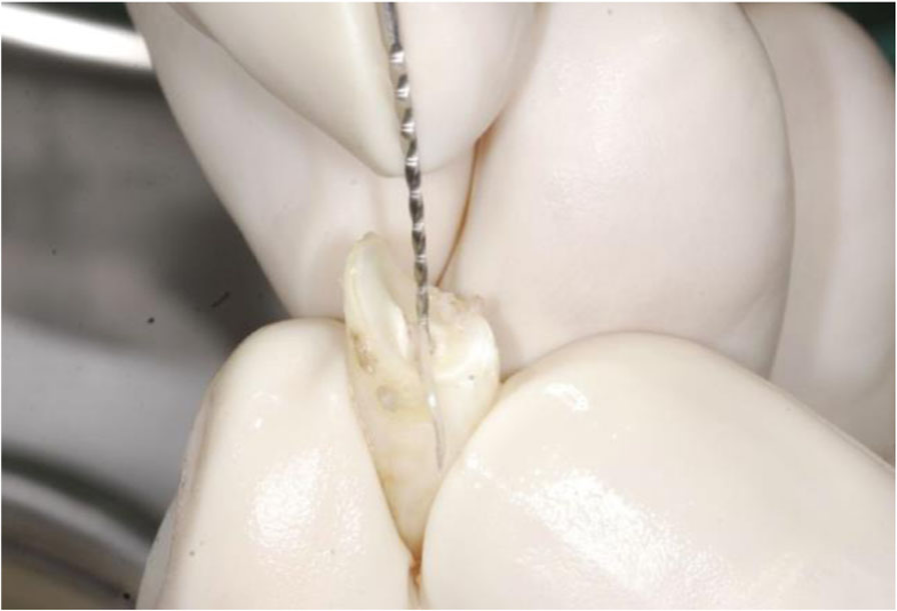
Removal of the pulp

**Fig. 4:**
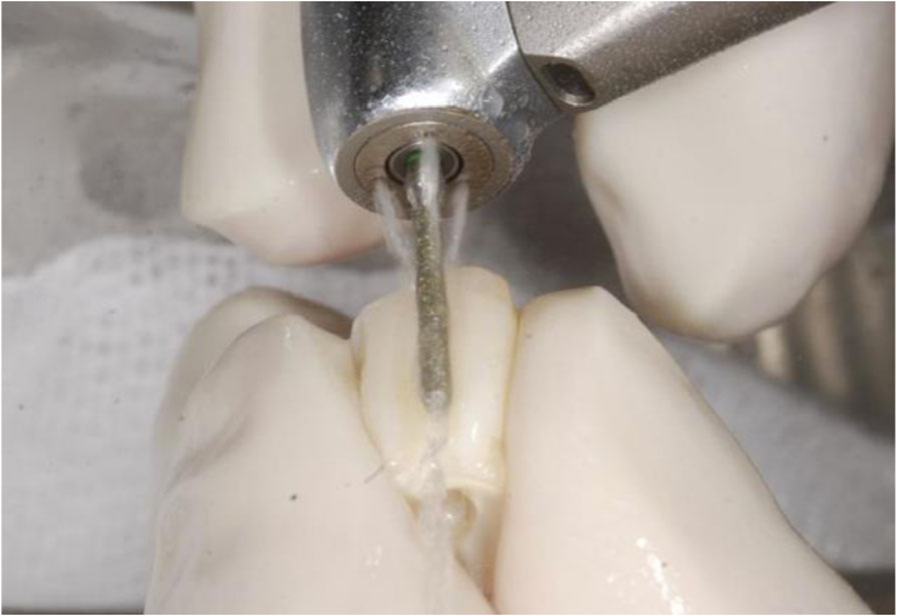
Removal of enamel and the cementum

The root surface was carefully cleaned from periodontal tissue. The pulp was removed, using a root canal instrument (K-file, Dema Dent AG, Bassersdorf, Switzerland). Layers of enamel and cementum were removed, using a rotating instrument (Diamond polisher, Rodent AG, Montlingen, Switzerland) (Figs. 3 and 4).

**Fig. 5:**
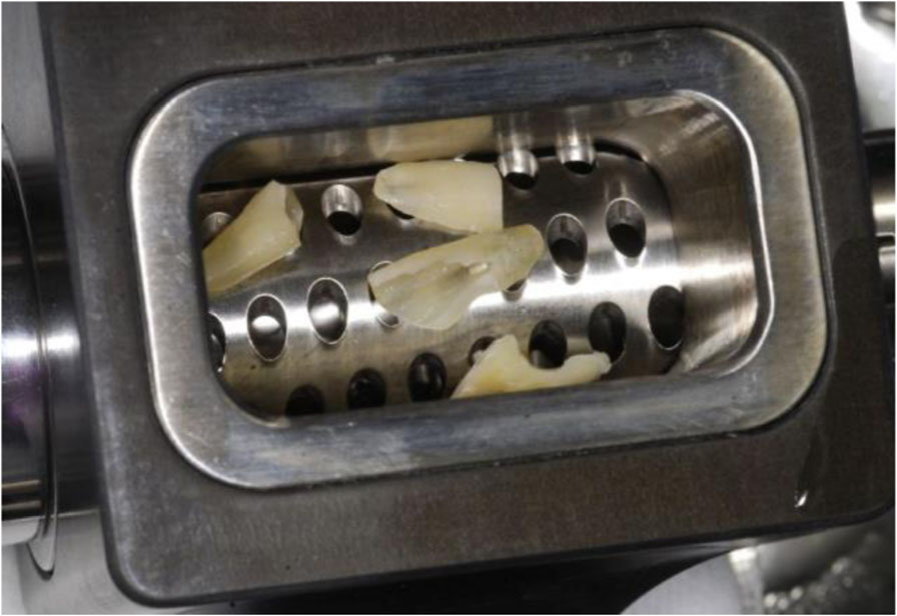
Autologous dentin in a bone mill

**Fig. 6:**
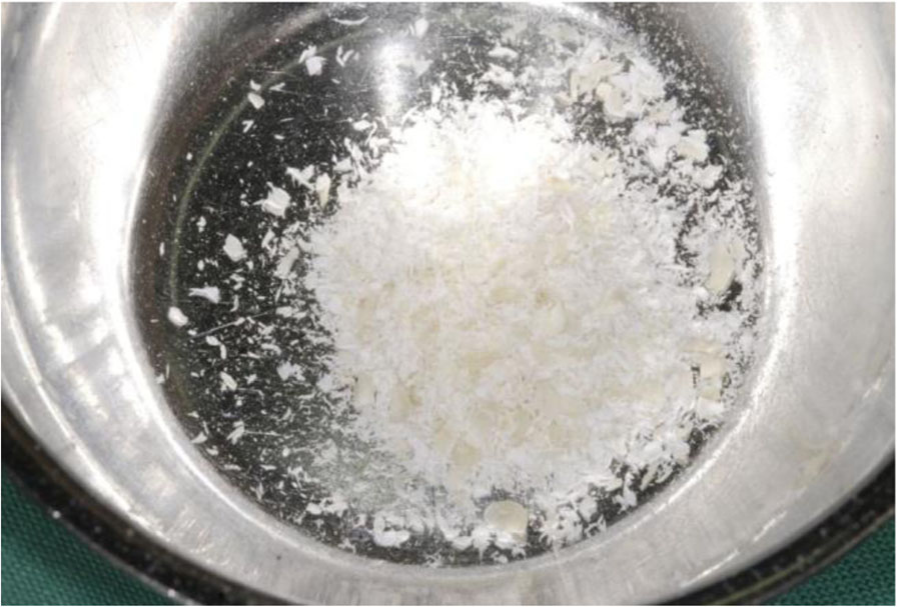
Autologous dentin with the desired particle size

Subsequently, the remaining dentin was cut into pieces (Bone rongeur forceps, Carl Martin BmbH, Solingen, Germany). These pieces of dentin were grinded using a bone mill (USTOMED INSTRUMENTE, Ulrich Storz GmbH & Co., Tuttingen, Germany) in order to achieve a particle size between 0.25 and 2 mm (Figs. 5 and 6).

**Fig. 7:**
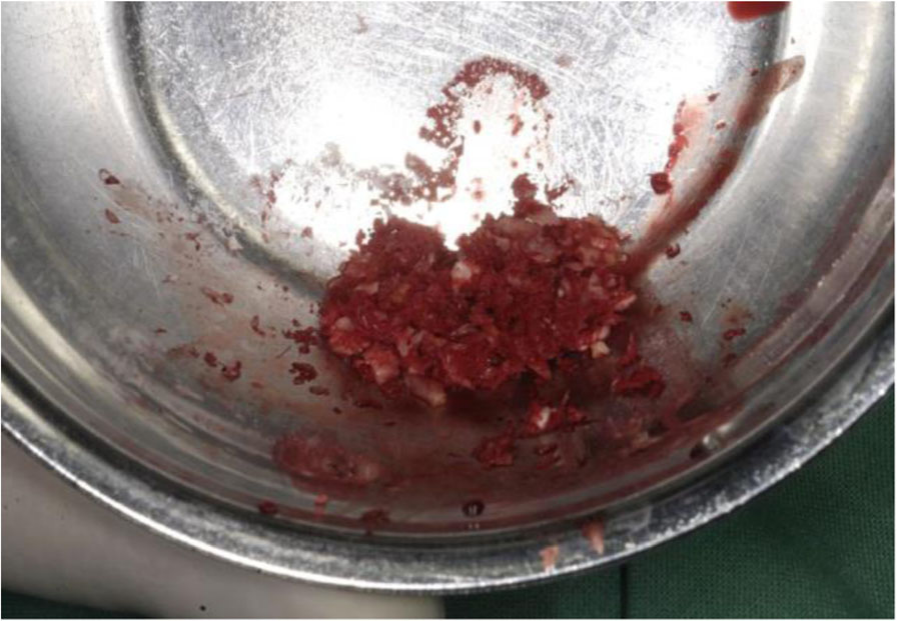
Autologous, particulated dentin mixed with blood from the operating site

**Fig. 8:**
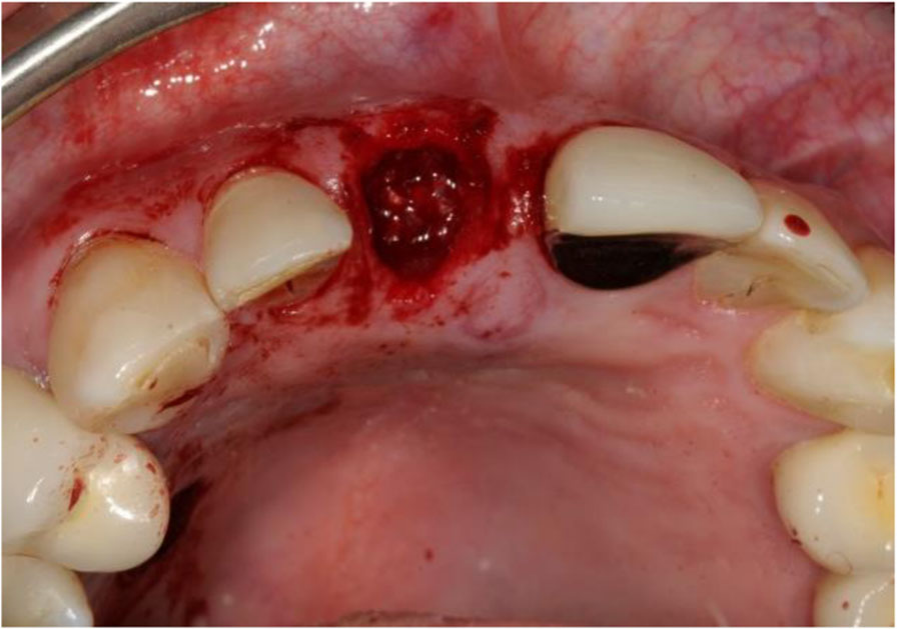
Autologous, particulated dentin in the alveolar socket

The autologous, particulated dentin was mixed with autogenous blood from the operating site (Fig. 7) and carefully inserted into the alveolar socket under controlled pressure to the level of the palatal/vestibular bone plate (Fig. 8).

**Fig. 9:**
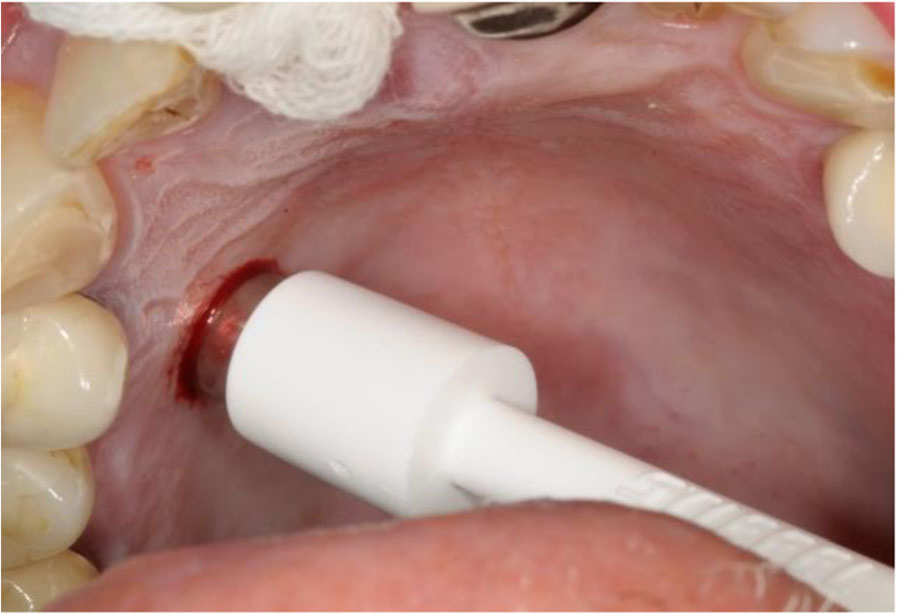
Soft tissue punch

**Fig. 10:**
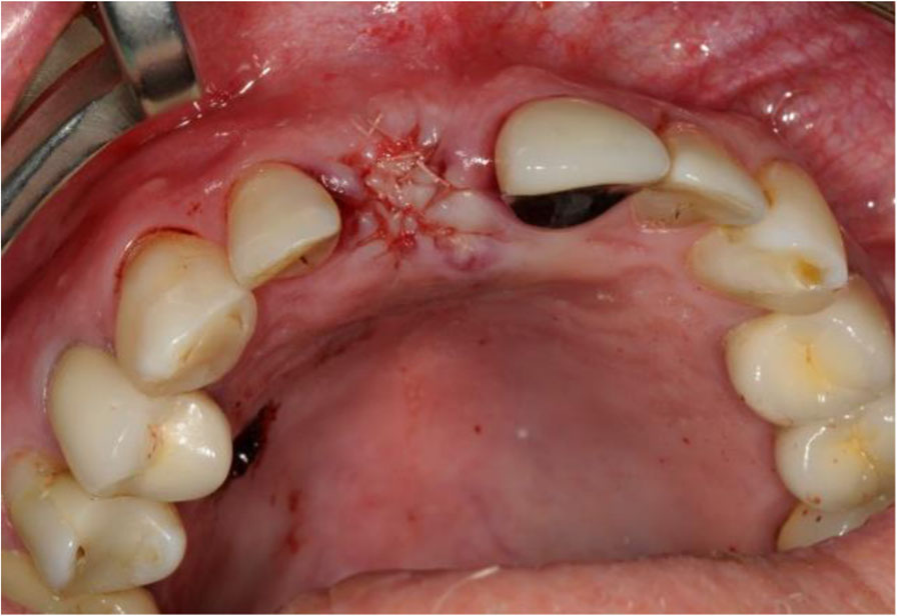
Soft tissue graft placed on the recipient site

An autologous soft tissue graft was harvested from the patient’s palate using a soft tissue punch (Biopsy Punch, kai Europe GmbH, Solingen, Germany) (Fig. 9). The graft had a comparable dimension as the recipient site. The gingival graft was placed on top of the augmentation material, adapted and carefully sutured to the marginal gingiva after the sulcus epithelium was removed with a rotating diamond (Vicryl 6-0, Ermed AG, Schleithem, Switzerland) (Fig. 10).

**Fig. 11:**
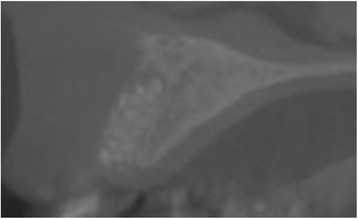
Sagittal view

**Fig. 12:**
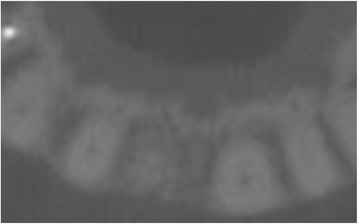
Axial view

In order to evaluate the ridge preservation properly, a cone beam computed tomography (CBCT, 3D Accuitomo, J. Morita Mfg. Corp., Kyoto, Japan) was taken post-surgery with a resolution of 0.25 mm (scan time 17.5 s, 90 kV, 5 mA). The findings were assessed on a computer (HP Compaq 6200 Pro Microtower PC, graphics card: Intel HD Graphics 2000 Dynamic Video Memory Technology, mouse: HP Compaq DC 172B; Hewlett Packard, Palo Alto, CA, USA) with a calibrated monitor (HP Compaq LA 2306x; Hewlett Packard, Palo Alto, CA, USA) using the reconstruction software Morita version I Dixel (J. Morita Mfg. Corp., Kyoto, Japan) (Figs. 11 and 12).

The patients received antibiotics peri-operatively and for 7 days post-surgery (Amoxicillin® 750 mg 1-1-1).

**Fig. 13:**
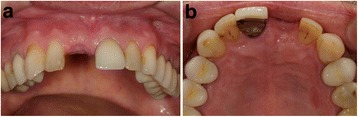
**a**, **b** Clinical situation prior to implant placement

The first follow-up consultation was 7 days post-surgery. The patients did not report any discomfort, and wound healing was regular in all four cases. No clinical signs of significant infection or graft loss were present. The sutures were removed 14 days post-surgery. Consecutive follow-up examinations did not show any complications, and implant placement was performed after 3 to 4 months (Fig. 13a, b).

The height and width of the ridge were sufficient prior to implant placement, which left at least 2 mm of buccal bone after implant placement.

## Case presentation

**Fig. 14:**
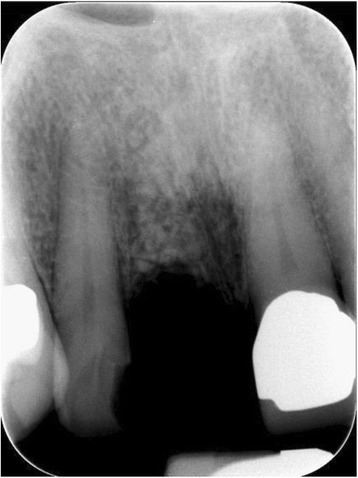
Single tooth X-ray immediately after the augmentation using autogenous dentin

**Fig. 15:**
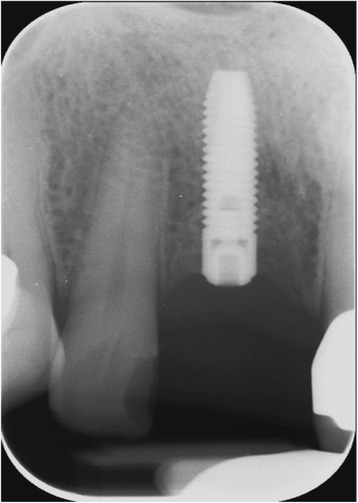
Single tooth X-ray, showing a constant bone level 7 months after implant placement

**Fig. 16:**
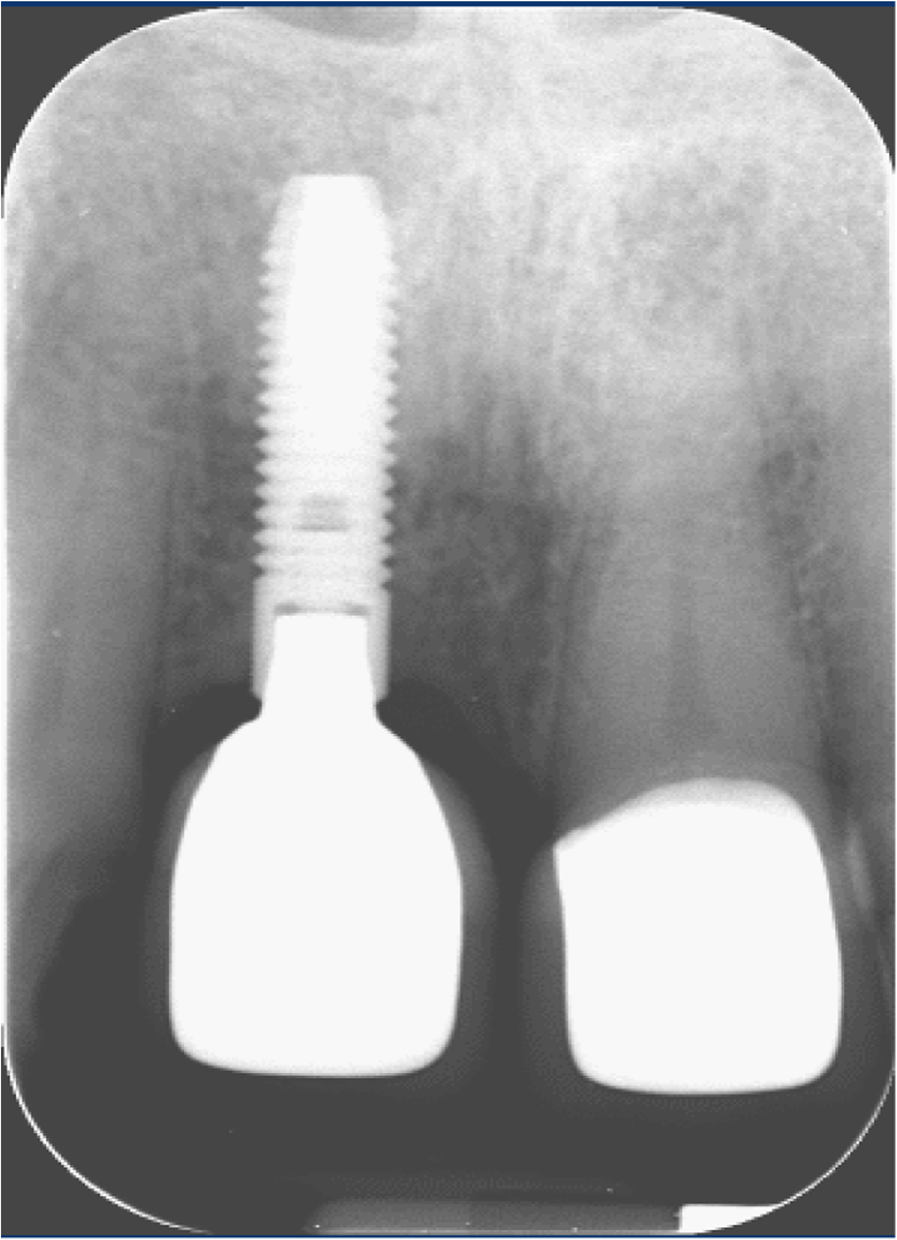
Single tooth X-ray, 1 year post-implantation, showing the finalized crown

The 1-year follow-up examination of the presented case showed an implant success, according to the appropriate clinical criteria [2] (Figs. 14, 15 and 16).

The pink esthetic score (PES) was used for the evaluation of reproducible soft tissue around the final implant crown as a parameter for the aesthetic outcome [12]. Seven variables were evaluated comparing the soft tissue around the implant with the neighbouring reference tooth. Using a 0-1-2 scoring system, the mesial papilla, distal papilla, soft tissue level, soft tissue contour, alveolar process deficiency, soft tissue colour and texture were evaluated.

The situation before the extraction of the tooth was scanned with an intraoral scanner (CEREC Omnicam®, Sirona-Dentsply, Bensheim, Germany), also the situation after the finalized prosthodontic restoration. The scans were superimposed, and the difference of the vertical and horizontal dimensions was calculated with specialized analysis software (Oracheck, Cyfex, Zurich, Switzerland).

## Results

Four months post-extraction and augmentation with autologous, particulated dentin, all four patients received an implant placement in the augmented area. In all cases, a CBCT was taken in between the dentin augmentation and the implant placement.

**Fig. 17:**
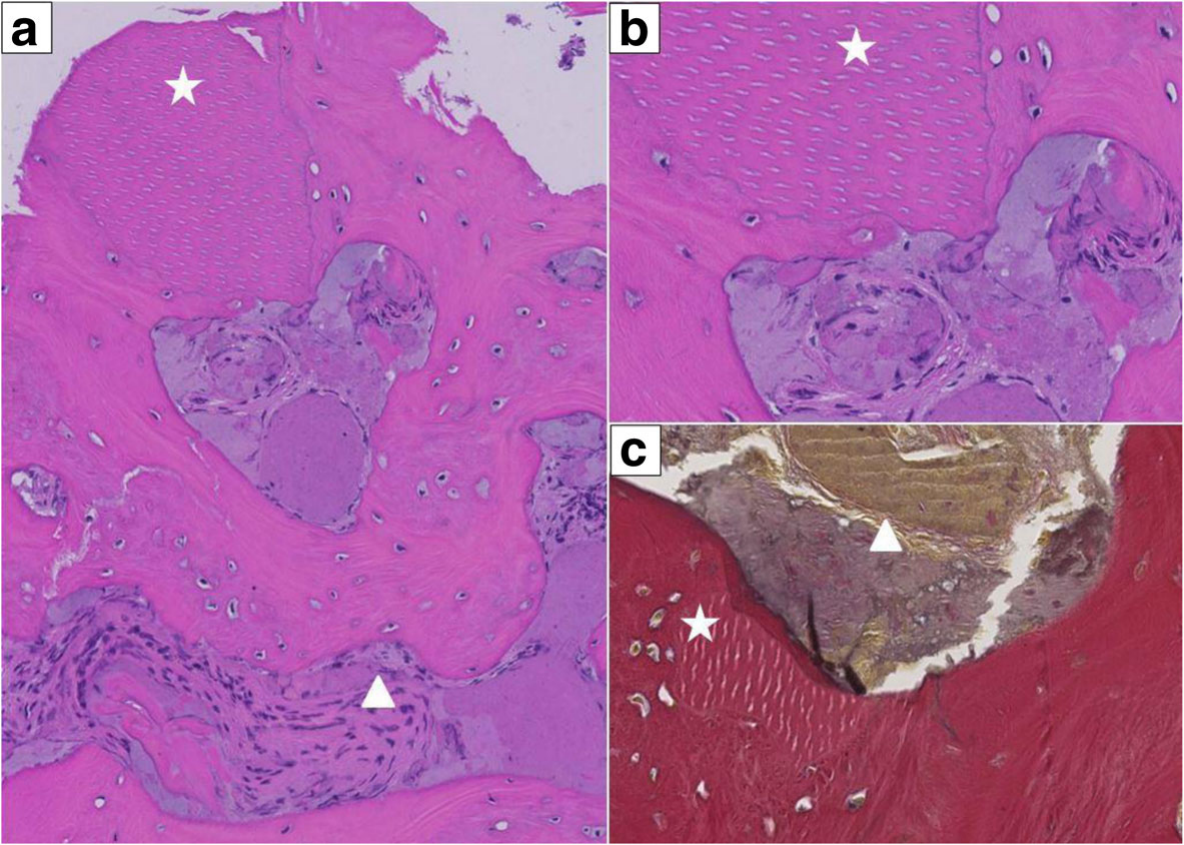
Histology of dentin augmentation. **a** *Asterisk* denotes incorporated dentin particle, surrounded by vital woven bone. *Triangle* shows reactive process in the bone marrow lacunae with osteoblast rimming. No signs of necrosis or infection (H&E stain, ×100 magnification). **b** Larger magnification at ×200. **c** EvG (Elastica van Gieson) stain, ×200

During implant placement, a biopsy of the bone from the augmented area was taken for histological examination (Fig. 17).

**Fig. 18:**
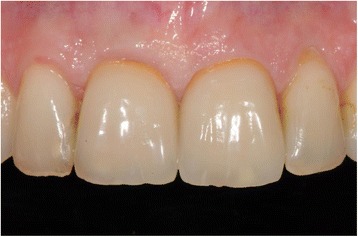
Finalized prosthetic restoration after 1 year

The final prosthetic solution demonstrated a functional and esthetical success of the used treatment method (Fig. 18).

In the presented case, a PES of 13 was evaluated, deducting one point for the soft tissue level under critical observation.

**Fig. 19:**
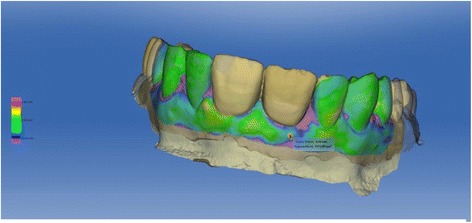
Colour-coded superimposition of intraoral scans before extraction and after definitive prosthetic restoration

**Fig. 20:**
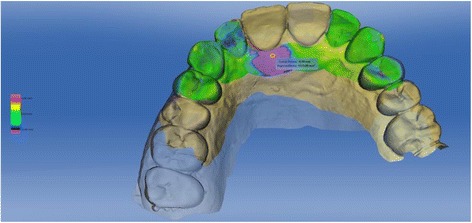
Colour-coded superimposition of intraoral scans before extraction and after definitive prosthetic restoration

A loss of 0.76 mm in the vertical dimension and a loss of 1.1 mm in the horizontal dimension could be observed in the calculation of the superimposed situations before extraction and 1 year after finalized prosthetic restoration (Figs. 19 and 20).

## Discussion

The aim of this case series is to demonstrate the efficacy and safety of this novel augmentative procedure for ridge preservation prior to implant therapy. This shall serve as a basis for a prospective study.

In all four cases, patients showed a stable volume of soft and hard tissues after the augmentation with AutoPD and good osseointegration of titanium implants, having been placed in this augmented socket.

The application of autologous bone and xenogeneic biomaterials for alveolar bone augmentation following tooth extraction has been intensively studied. This so called ridge preservation aims at the prevention of bone atrophy. From a biological point of view, autologous bone is still considered to be the optimal augmentation material due to its osteogenic, osteoinductive and osteoconductive properties [1, 34]. However, especially in small defects, possible donor-site morbidity, limited graft volume availability and additional length of operation for harvesting autologous bone led to the increasing usage of xenogeneic biomaterials such as demineralized bovine bone substitute (DBBS—Bio-Oss^©^). These kind of non-resorbable biomaterials have great potential in maintaining the dimension of the contour of the ridge by serving as a framework for new bone formation [7]. Although DBBS shows great osteoconductive potential and has been proven to be as effective as autologous bone alone or in combination with autologous bone, it has a slow and incomplete resorption rate [4, 14, 22, 24].

In addition, the use of DBBS increases treatment cost and may be incompatible to some patients. Regarding these factors of influence, it is of interest to test alternative bone substitute materials.

In traumatology, many studies showed that replanted teeth with a devitalized periodontal tissue will ankylose and dentin will be replaced by bone [1, 3].

It is well known that dentin and bone have a similar organic and inorganic structure [21]. Recent studies have focused on dentin as a potential bone substitute in different models of alveolar defects. It could be shown that dentin, being used either as a block graft or in particulated form, is involved in bone remodelling, expressing osteoconductive and even osteoinductive properties [3, 5, 9, 26, 29, 30]. In vivo studies in mice showed that dentin scaffolds performed similar with regard to the inflammatory response and neovascularization compared to isogenic bone [9]. Both materials induced an acute short-term inflammatory response with increased leukocyte-endothelial cell interaction, a process often observed after the implantation of biomaterials [19, 27]. Additionally, in vitro studies showed that protein extracts from dentin affect proliferation and differentiation of osteoprogenitor cells. Results suggested that TGFβ and perhaps other factors in dentin can regulate cell behaviour and, therefore, can influence development, remodelling and regeneration of mineralized tissues [33].

In humans, particulated tooth material has been used for sinus augmentation in order to enhance implant therapy. Preliminary results from five patients histologically showed an osteoconductive osteogenesis with partial resorption of tooth components [25].

In the present case series, all patients underwent socket preservation with AutoPD. In all cases, one or two upper frontal central incisors were extracted. The teeth were immediately removed of the pulp or root canal filling, enamel and cementum. AutoPD enriched by autogenous blood was inserted into the alveolar socket without a further chemical modification or sterilization process during the same operation. In a recent study, Pang et al. used a demineralized autologous dentin matrix for socket preservation, however 2 to 4 weeks after tooth extraction. Additionally, the dentin matrix was sterilized before the augmentation process [23]. This procedure should potentially reduce the risk of inflammation but demands a second surgical intervention. It is currently unknown, whether such a procedure is necessary.

In the present experimental treatment concept, it has to be emphasized that the extraction was performed as atraumatic as possible. In all cases, the buccal lamella was intact prior to augmentation of AutoPD and a flapless approach had been chosen. After augmentation, the socket was covered by a patch, harvested from the palate with the punch technique. Wound healing was uneventful for all patients. In one case, a histological probe has been gained after 4 months during implant placement. The histological examination showed evidence of remodelling processes between dentin and bone without any signs of inflammation.

## Conclusion

Within the limits of this case series, it has been shown that particulated dentin of autologous teeth may serve as an alternative to autologous bone for alveolar ridge preservation prior to implant therapy. However, randomized studies on this treatment option are necessary.
